# Alternative methods for RuBisCO extraction from sugar beet waste: A comparative approach of ultrasound and high voltage electrical discharge

**DOI:** 10.1016/j.ultsonch.2023.106535

**Published:** 2023-07-27

**Authors:** Josipa Dukić, Karla Košpić, Vanja Kelava, Renata Mavrić, Marinela Nutrizio, Biljana Balen, Ana Butorac, Mecit Halil Öztop, Anet Režek Jambrak

**Affiliations:** a Faculty of Food Technology and Biotechnology, University of Zagreb, 10000 Zagreb, Croatia; b Department of Biology, Faculty of Science, University of Zagreb, 10000 Zagreb, Croatia; c BICRO BIOCentre Ltd, Cent Lab, 10000 Zagreb, Croatia; d Department of Food Engineering, Middle East Technical University, 06800 Ankara, Turkey

**Keywords:** Zero waste concept, Ultrasound, High voltage electrical discharge, Sugar beet leaves, Protein extraction, RuBisCO

## Abstract

Ultrasound (US) and high voltage electric discharge (HVED) with water as a green solvent represent promising novel non-thermal techniques for protein extraction from sugar beet (*Beta vulgaris* subsp. *vulgaris* var. *altissima*) leaves. Compared to HVED, US proved to be a better alternative method for total soluble protein extraction with the aim of obtaining high yield of ribulose-1,5-bisphosphate carboxylase-oxygenase enzyme (RuBisCO). Regardless of the solvent temperature, the highest protein yields were observed at 100% amplitude and 9 min treatment time (84.60 ± 3.98 mg/g_d.m._ with cold and 96.75 ± 4.30 mg/g_d.m__._ with room temperature deionized water). US treatments at 75% amplitude and 9 min treatment time showed the highest abundance of RuBisCO obtained by immunoblotting assay. The highest protein yields recorded among HVED-treated samples were observed at a voltage of 20 kV and a treatment time of 3 min, disregarding the used gas (33.33 ± 1.06 mg/g_d.m__._ with argon and 34.89 ± 1.59 mg/g_d.m._ with nitrogen as injected gas), while the highest abundance of the RuBisCO among HVED-treated samples was noticed at 25 kV voltage and 3 min treatment time. By optimizing the US and HVED parameters, it is possible to affect the solubility and improve the isolation of RuBisCO, which could then be purified and implemented into new or already existing functional products.

## Highlights

• Waste utilization is in line with sustainable goals.
• Sugar beet leaf, a waste from sugar industry, is a good source of RuBisCO enzyme.
• US and HVED showed to be a good techniques for the extraction of Rubisco enzyme.
• Higher yield of RuBisCO enzyme is achieved by US.

## 1 Introduction

Nowadays, due to the rapid lifestyle and constant socioeconomic changes, the dietary habits of modern society are changing worldwide [1], [2]. Furthermore, with population growth, there is an increasing demand for cheaper and more sustainable plant protein sources to supplement or replace expensive and limited meat protein sources [3], [4], [5]. Specifically, to achieve sustainability and contribute to Agenda 2030, the emphasis is placed on reducing the consumption of animal proteins and increasing the consumption of proteins from plant sources [6], [7]. Products containing plant-based proteins can provide almost equal quality compared to meat and dairy products [5], [8], but at lower costs, while meeting the global priority of reducing greenhouse gas emissions and protecting the environment [9], [10]. Plants and algae could serve as valuable sources of proteins for the production of bioactive peptides, components that have beneficiary effects on human health [11]. So far, legumes such as soybeans [12], peas [13], and by-products of the vegetable oil industry have been mainly used as protein sources [14], [15], [16], but recently the use of leaves of green plants such as spinach and alfalfa has been gaining attention [17], [18]. In addition, sugar beet leaves left in beet fields after harvest are a potentially good source of proteins [19], which also provides economic benefits and contributes to sustainable development goals [20], [21], [22].

In the sugar industry, sugar beet leaves represent waste [23], which has recently been used in biotechnology as raw material in the production of bioethanol [24]. Considering the significant content of 25–35% protein in g of dry matter [25], [26], the valorization of leaves is increasingly being investigated for other purposes as well [19], [27]. In leaf cells, most of the soluble and insoluble proteins (70%) are located in chloroplasts [28], of which ribulose-1,5-bisphosphate carboxylase-oxygenase (RuBisCO) and the proteins of the chlorophyll/light binding complex are most abundant [29]. Under optimal conditions, RuBisCO accounts for nearly 50% of the soluble protein fraction in sugar beet leaves [30], [31], and its presence in the leaves of numerous plants and green algae makes it the most abundant protein on Earth [32]. In its native state, RuBisCO contains peptides with bioactive properties, which potentially may have a promising application in the development of new and improved functional products [33], [34], [35]. Furthermore, RuBisCO has been shown to have significant biological value due to its essential amino acid content. Specifically, it is rich in tryptophan, leucine, tyrosine, and phenylalanine [36], [37], [38]. In addition to high biological value, RuBisCO also has desirable physical properties such as gelling, foaming and emulsifying, which could enable food manufacturers to successfully incorporate them into numerous food products [36], [38], [39]. Despite its high occurrence in nature and its outstanding functionality, RuBisCO remains underutilized as a protein in industrially produced food, mainly due to the technological challenges associated with its extraction from plants and its low yield in terms of leaf mass, since 85–90% of the leaf consists of water [33]. In addition, extraction processes for obtaining protein isolates can greatly affect the solubility, degree of denaturation and composition of the product, which consequently changes the biological and physical properties of the final product [40]. Furthermore, it has been reported that the choice of extraction method may significantly impact the final concentration and recovery of proteins from sugar beet leaves [41], [42]. Extreme conditions such as high temperatures and pH can negatively affect protein functionality [43].

Therefore, the use of non-thermal, green extraction methods such as ultrasound (US) and high voltage electric discharge (HVED) are being examined. The application of US in the extraction of high-value bioactive compounds has been raised to the industrial level, whereas its application in protein extraction is still underexploited while protein extraction methods are mainly conducted on a laboratory scale [44]. Extraction assisted by ultrasound is a mechanical procedure based on sound waves of certain frequencies and amplitudes that accelerate mass transfer and heat, which increases the permeability of cell walls and membranes, promoting the release of cell content [45]. Oscillations and implosion of cavitation bubbles that arise as a result of the phenomenon of acoustic cavitation cause shock waves, micro-jets, turbulence, and shear forces that result in fragmentation, erosion, sonoporation, and destruction of cell structures [46]. Due to changes in the secondary and tertiary structure of proteins and better exposure of hydrophilic groups of amino acids to the solvent, the solubility of proteins increases [46], [47]. For all these reasons, there is an aspiration to raise ultrasound protein extraction from laboratory to industrial level. On the other hand, HVED has been mainly exploited for food-contact surfaces disinfection [48], water disinfection [49], and enzyme inactivation [50], but recently, scientific research has increasingly focused on the application of HVED for the extraction of bioactive components from various raw materials [51], [52], [53]. The method itself is based on an electric discharge between two electrodes due to high voltage, which leads to the formation of a cold plasma [54]. When living cells are placed in an electric field, the pores of the cell membranes open, the so-called phenomenon of electroporation, in which biomolecules are released from the cells [55]. Non-thermal techniques such as HVED enable increasing the speed and yield of extraction of bioactive components from plant material with minimal energy consumption [56]. For this method to be successful in achieving the highest possible protein yield, optimal extraction conditions, i.e., voltage, temperature, and treatment time, must be ensured with respect to the protein source and the target component for extraction [57].

In this study, the efficacy of US and HVED treatments on the extraction of total soluble proteins from sugar beet leaves was evaluated by varying the applied amplitude and treatment time for US treatments and the applied gas, applied voltage, and treatment time for HVED treatments. The aim was to obtain a high RuBisCO yield with respect to leaf mass, which could then be used as a source of bioactive peptides for functional food production. The emphasis is put on sustainability, using the industrial waste material as a protein source, in combination with an extraction method with low environmental impact.

## 2 Materials and methods

### 2.1 Chemicals

Most chemicals were purchased from Sigma-Aldrich (St. Louis, MO, USA). Chemicals from other manufacturers are listed below: argon (Messer Croatia Plin, Zaprešić, Croatia), nitrogen (Messer Croatia Plin, Zaprešić, Croatia), MilliQ water (BIOCentar, Zagreb, Croatia), trichloroacetic acid (Merck & Co., Rahway, NJ, USA), acetonitrile (VWR Chemicals, Radnor, PA, USA), formic acid (VWR Chemicals, Radnor, PA, USA), tetrahydrofuran (J.T.Baker, Phillipsburg, NJ USA), 1 M triethylammonium bicarbonate (Thermo Fisher Scientific, Waltham, MA, USA), trypsin (Promega, Madison, WI, USA), Glu-C (Promega, Madison, WI, USA), TFQGPPHGIQVER (Thermo Fisher Scientific, Waltham, MA, USA), AQAETGEIK (Thermo Fisher Scientific, Waltham, MA, USA), acetone (VWR Chemicals, Radnor, PA, USA), Tris(2-carboxyethyl)phosphine hydrochloride (Thermo Scientific, Waltham, MA, USA).

### 2.2 Plant materials

Sugar beet (*Beta vulgaris* subsp. *vulgaris* var. *altissima*) leaves were provided by project partners from Turkey (Kayseri Şeker, Kocasinan Kayseri, Turkey). To facilitate extraction during sample preparation, the leaves were grinded to plant particle size distribution of d(0.1) ≤ 238.490 µm; d(0.5) ≤ 630.116 µm; d(0.9) ≤ 1196.769 µm measured by the laser particle size analyzer Mastersizer 2000 (Malvern Instruments GmbH, Herrenberg, Germany).

### 2.3 Labeling of samples and extraction

#### 2.3.1 Sample labels

LC – Sugar beet leaves extracted by phenolic extraction method (control sample).

LUDI – Sugar beet leaves US-treated (room temperature deionized water).

LUDW – Sugar beet leaves US-treated (cold deionized water).

LHA – Sugar beet leaves HVED-treated with argon as injected gas.

LHN – Sugar beet leaves HVED-treated with nitrogen as injected gas.

In addition to these basic labels, a numerical designation was added to the US and HVED treated samples as listed in Table 1**.**

**Table 1:** Numerical designations and process parameters for US and HVED treated samples.

Sample	Amplitude (%)	Treatment time (min)	Voltage (kV)	Applied gas	Solvent temperature (°C)
LUDI1	75	6	/	/	25
LUDW1					4
LUDI2	75	3	/	/	25
LUDW2					4
LUDI3	50	6	/	/	25
LUDW3					4
LUDI4	50	9	/	/	25
LUDW4					4
LUDI5	75	9	/	/	25
LUDW5					4
LUDI6	100	9	/	/	25
LUDW6					4
LUDI7	50	3	/	/	25
LUDW7					4
LUDI8	100	6	/	/	25
LUDW8					4
LUDI9	100	3	/	/	25
LUDW9					4
LHA1	/	6	25	Argon	25
LHN1				Nitrogen	
LHA2	/	6	20	Argon	25
LHN2				Nitrogen	
LHA3	/	9	20	Argon	25
LHN3				Nitrogen	
LHA4	/	9	25	Argon	25
LHN4				Nitrogen	
LHA5	/	3	20	Argon	25
LHN5				Nitrogen	
LHA6	/	3	25	Argon	25
LHN6				Nitrogen	

#### 2.3.2 Protein extraction

All ultrasonic extractions were performed using Q700 Sonicator (Qsonica, Newtown, CT, USA), by adding 100 mL of deionized water (cold, 4 °C or room temperature, 22 °C) into a 250 mL laboratory beaker with 2 ± 0.001 g of weighed crushed sugar beet leaves. During extractions, an ultrasound probe (diameter 12 mm) was placed in the center of the laboratory beaker and immersed in the liquid for about 2.4 cm, sufficiently spaced from the bottom, as previously described in Dukić et al. [19]. Furthermore, all US treatments were performed according to previously optimized extraction parameters: the amplitude of 50, 75, and 100%, and treatment time of 3, 6, and 9 min. To prevent overheating, laboratory beakers were placed into a plastic container with ice cubes and water. Comparative, high-voltage electric discharge extractions were performed in a 100 mL reactor. A total of 1 ± 0.001 g of dry sugar beet leaf samples were added to 50 mL of distilled water at room temperature, as extracting solvent. For electric discharge, IMP-SSPG-1200 generator (Impel group d.o.o., Zagreb, Croatia) was used and previously described in more detail by Nutrizio et al. [58]. The treatments were performed according to previously optimized extraction parameters: frequency of 100 Hz, voltage of 15 and 20 kV for argon gas, and 20 and 25 kV for nitrogen gas, a pulse width of 400 ns, and treatment time of 3, 6, and 9 min. The gap between electrodes was 15 mm. Furthermore, argon and nitrogen gases were flowed in through the needle with a flow of 0.75 L/min. Extracts obtained by US and HVED treatments were filtered using a Büchner funnel and analyzed. The control sample was extracted using the phenol extraction method according to Faurobert et al. with minor modifications [59]. Namely, 0.05 g of dry leaves was extracted in 4 mL of the extraction buffer [500 mM Tris, 50 mM EDTA, 700 mM sucrose, 100 mM potassium chloride (KCl), 1 mM phenylmethylsulfonyl fluoride (PMSF), and 2% β-mercaptoethanol], incubated on ice for 10 min and extracted in 4 mL of phenol. After centrifugation (10 min, 20000 × g) the supernatant phenolic phase was extracted and mixed with 4 mL of extraction, incubated on ice for 3 min, and centrifugated (10 min, 20000 × g). Protein precipitation from the supernatant phase was done overnight at −20 °C using ice-cold precipitation solution [0.1 M ammonium acetate (NH_4_CH_3_CO_2_) in methanol], and obtained pellets were washed 3 × with 3 mL of ice-cold precipitation solution and 1 × with 3 mL of ice-cold acetone and dissolved in 500 μL of isoelectric focusing (IEF) buffer [9 M urea and 4% (*w/v*) 3-((3-cholamidopropyl) dimethylammonio)-1-propanesulfonate (CHAPS)] supplemented with 2 mg/mL of dithiothreitol (DTT) and 0.52% (*v/v*) of ampholytes.

### 2.4 Analysis

#### 2.4.1 Determination of total protein content

Protein concentration from the samples was measured by UV/Vis spectrophotometer Specord 50 PLUS (Analytik Jena, Jena, Germany) according to the Bradford method [60] using bovine serum albumin (BSA) as a standard, and calculated using linear equation:

(1) y=0.3971x-0.08

where “*y*” represents a measured absorbance (at 595 nm) and “*x*” equivalent BSA concentration (in mg/mL).

Calibration curve is shown in Fig. S1 (Supplementary material). Considering the known mass of sugar beet leaf and the proportion of dry matter (94.49 ± 1.6%), the results were expressed in mg/g_d.m._. Determination of dry matter was performed by drying to constant weight as described in Dukić et al [19].

#### 2.4.2 RuBisCO immunodetection by western blotting

Protein extracts were mixed with a modified Laemlli sample buffer (4x) [61] with the addition of 250 mM DTT, and denatured on 95 °C for 10 min. 3 µg/well of total protein were separated on 4–10% sodium dodecyl-sulfate polyacrylamide gel electrophoresis (SDS-PAGE) and blotted 1 h to nitrocellulose membrane (Cytiva Amersham™ Protran™ NC Nitrocellulose Membranes, pore size 0.2 µm), using wet transfer. Membrane was blocked with 2% milk in phosphate buffered saline (PBS) containing 137 mM NaCl, 2.7 mM KCl, 8 mM Na_2_HPO_4_, and 2 mM KH_2_PO_4,_ with the addition of 0.5 % Tween (PBS-T) for 1 h/RT. Blot was then probed with the primary antibody rabbit anti-RuBisCO large subunit purchased from Agrisera (Vannas, Sweden, AS03 037) at a dilution of 1:5000 for 1 h/RT with agitation in a solution of 2% milk in PBS-T and then left overnight at 4 °C. The antibody solution was decanted, and the blot was washed 3× for 10 min in 2% milk in PBS-T at RT with agitation. Blot was incubated in Agrisera matching secondary antibody (Goat anti-rabbit IgG, horseradish peroxidase conjugated, AS09 602) diluted to 1:20000 for 1 h/RT with agitation. The blot was washed twice for 10 min in PBS-T and developed for 5 min with AgriseraECL Bright (AS16 ECL-N-10). Exposure time was 12 min, and the hybridization images were obtained by LI-COR C® Digit Blot scanner (LI-COR Biosciences – GmbH, Bad Homburg vor der Höhe, Germany). For band weight prediction, prestained molecular weight marker - ColorBurstTM Electrophoresis Marker Sigma (C1992-1VL) was used.

#### 2.4.3 RuBisCO quantification

##### 2.4.3.1 Sample preparation

The samples were diluted with MilliQ water to a final protein concentration of 0.17 mg/mL. Samples were prepared in two sets. One set of samples was used for the determination of RuBisCO concentration, and the other one was used to simulate the matrix effect of a calibration curve. 10 µL of 1 mg/mL BSA (internal standard) was added to each sample, then the samples were precipitated after the addition of an aqueous 7.5% trichloroacetic acid solution (*w/v*) and 0.1% sodium deoxycholate (*w/v*), as described by Chevallet et al. [62]. Digestion was performed with trypsin (final trypsin concentration was 0.02 mg/mL) at 37 °C, 600 rpm, overnight. The samples were filtered through a 0.2 µm filter, diluted 1:1 (*v/v*) with MilliQ water, and analyzed. Preparation of the samples for stimulating the matrix effect was carried out as described above, however, the digestion was carried out with Glu-C enzyme (the final concentration of Glu-C was 0.02 mg/mL) at 37 °C and 600 rpm, overnight. The samples were filtered through a 0.2 µm filter, diluted 1:1 (*v/v*) with MilliQ water, and 5 µL of BSA tryptic digest was added as an internal standard. TFQGPPHGIQVER and AQAETGEIK peptides (purity > 98%) ordered from Thermo Scientific were used for calibration curve preparation as shown in Fig. S2 and S3 (Supplementary material). The range of calibration curves was set from 100 to 5000 ng/mL. Linear equations for peptides concentrations are listed below:

(2) y=0.085753x-2.441085

where “*y*” represents relative response and “*x*” TFQGPPHGIQVER peptide concentration (in ng/mL).

(3) y=0.035212x+0.410465

where “*y*” represents relative response and “*x*” AQAETGEIK peptide concentration (in ng/mL).

##### 2.4.3.2 Liquid chromatography coupled with mass spectrometry

The multiple reaction monitoring (MRM) method for RuBisCO quantification was created and optimized using Skyline version 21.2.0.425 [63]. The RuBisCO protein sequence was downloaded from the UniProt database, accession number A0A023ZPS4. *Beta vulgaris* subsp. *vulgaris* proteome was used as background proteome (9803 entries, UniProt database, accessed 05.01.2022), the unique peptides of the target protein were selected for method development and sample analysis (TFQGPPHGIQVER and AQAETGEIK).

Method development and sample analysis were performed using a 6460 Triple Quad LC/MS system (Agilent technologies, Santa Clara, CA, USA) equipped with an electrospray ionization source. Chromatographic separation was performed at a constant flow rate of 0.4 mL/min on the Waters ACQUITY UPLC BEH C18 1.7 µm, 2.1 × 150 mm. The injection volume was 4 µL. Mobile phase compositions were 0.1% formic acid in water (*v/v*) and 0.1% formic acid in acetonitrile (*v/v*), the run time of the method was set to 15 min. Peptides were eluted at a flow rate of 0.4 mL/min with elution profile: 0 – 2 min: 2% B, 2 – 10 min 2 – 60% B, 10 – 11 min: 60 – 90% B, 11 – 13 min: 90% B, 13 – 14 min: 90 – 2% B and 14 – 15 min: 2% B. The MS was operated in positive electrospray ionization mode, a capillary voltage was set to 3.5 kV, gas temperature 300 °C, gas flow rate 7 L/min, nebulizer 40 psi, sheath gas temperature 300 °C, sheath gas flow rate 9 L/min. The MRM transition list is given in Table 2. The spectra were analyzed using Agilent MassHunter Workstation software (Agilent technologies, Santa Clara, CA, USA). An internal standard method (using spiked BSA) was employed for normalization where **bold** transitions were used for quantification.

**Table 2:** MRM transitions used for detection.

Peptide	Precursor	Product	Fragmentor (V)	Collision energy (eV)	Polarity
LGEYGFQNALIVR(IS BSA)	**740.4**	**813.5**	**130**	**24.0**	**Positive**
	740.4	1017.6	130	24.0	Positive
	740.4	813.5	130	24.0	Positive
TFQGPPHGIQVER	489.1	544.9	130	10.0	Positive
	489.1	600.4	130	12.0	Positive
	489.1	220.9	130	14.0	Positive
AQAETGEIK	**473.7**	**747.4**	**130**	**15.7**	**Positive**
	473.7	676.4	130	15.7	Positive
	473.7	446.3	130	15.7	Positive

#### 2.4.4 pH

After the US and HVED extractions, the pH value of the samples was measured using a pH-EC meter HI5521-02 (Hanna Instruments Inc., Zagreb, Croatia).

#### 2.4.5 Experimental design and statistical analysis

Experimental design and statistical analysis of US and HVED extraction parameters were performed in the STATGRAPHICS Centurion (StatPoint Technologies, Inc, Warrenton, VA, USA). The experiment included 2 groups of 9 US samples (LUDI1-9 and LUDW1-9) and 2 groups of 6 HVED samples (LHA1-6, and LHN1-6). Multilevel Factorial Design was used to determine the potential impact of input (independent) variables on output (dependent) variable. The independent parameters of the experiment for US-treated samples were applied amplitude (50, 75, and 100%) and treatment time (3, 6, and 9 min). Furthermore, for HVED-treated samples, independent parameters were applied voltage (20 kV and 25 kV) and treatment time (3, 6, and 9 min). Onwards, for US and HVED-treated samples, total protein content [mg/mL BSA] represented the dependent variable. US and HVED parameters had a statistically significant effect if *p* < 0.05, indicating that they differ significantly from zero in the 95.0% confidence interval.

## 3 Results

### 3.1 Total soluble protein content

#### 3.1.1 Protein yield after US extraction

**Fig. 1:**
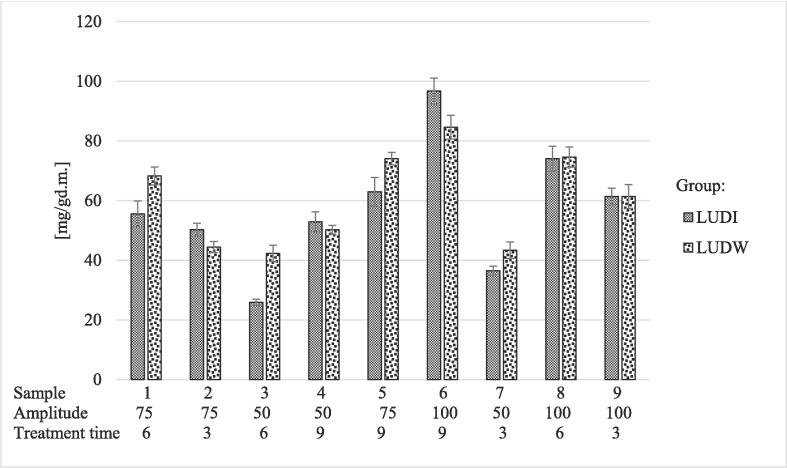
Protein yield for US-treated LUDI and LUDW samples. The applied amplitude (%) and treatment time (min) are listed below each sample. Results were presented as mean value of replicates ± standard deviation.

In the samples treated with US, a statistically significant influence of the amplitude and treatment time on the total protein yield was recorded (*p* < 0.05). In contrast, mutual interaction and individual quadratic interactions of treatment time and amplitude did not show a statistically significant influence on the yield of total proteins (*p >* 0.05) as shown in Table 3. The optimal extraction conditions were recorded at 100% amplitude and 9 min treatment time for both sample groups (LUDI/LUDW). These are also the parameters where the highest yield of total proteins was recorded, specifically, 84.60 ± 3.98 mg/g_d.m__._ in LUDW6 and 96.75 ± 4.30 mg/g_d.m__._ in LUDI6 samples. In contrast, the lowest yields were recorded for LUDW3 (42.28 ± 2.73 mg/g_d.m._) and LUDI3 (25.91 ± 1.02 mg/g_d.m._) samples. Furthermore, the most substantial difference in total protein yield (16.37 mg/g_d.m._) among the two groups of samples was observed between LUDW3 and LUDI3 samples. These results are presented graphically in Fig. 1. Compared to the control sample (254.67 ± 8.85 mg/g_d.m._), 2.63 – 9.83 times lower yield of total proteins was observed in the US-treated samples.

**Table 3:** Statistical significance for protein yield. MANOVA statistically processes the variability of each input parameter, their mutual interactions, and quadratic interactions of amplitude and treatment time on the output values of LUDI and LUDW samples.

Sample group		Main effects		Interactions		
		A: Amplitude	B: Treatment time	AA	AB	BB
*p*	LUDI	**0.00**	**0.04**	0.78	0.30	0.22
	LUDW	**0.01**	**0.03**	0.59	0.30	0.69

Where A determines Amplitude and B stands for Treatment time. The *p-*values < 0.05, indicating that they are significantly different from zero at the 95.0% confidence level.

#### 3.1.2 Protein yield after HVED extraction

**Fig. 2:**
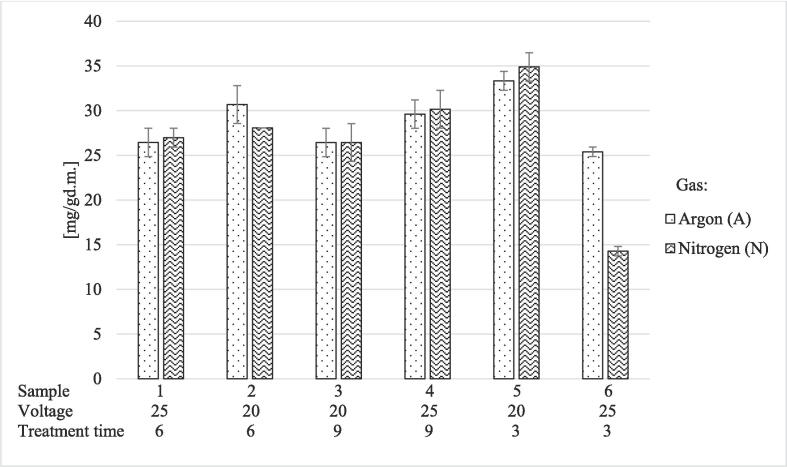
Protein yield for HVED-treated LHA and LHN samples. The applied voltages (kV) and treatment time (min) are listed below each sample. Results were presented as mean value of replicates ± standard deviation.

The results of protein yield obtained after the HVED extraction are expressed according to g of dry matter (d.m.) and shown in Fig. 2. The highest yields of proteins were observed after 20 kV applied voltage and 3 min treatment time, regardless of the applied gas. Specifically, with applied argon, the highest amount of protein was observed in LHA5 (33.33 ± 1.06 mg/g_d.m._) and with nitrogen in LHN5 sample (34.89 ± 1.59 mg/g_d.m._). The lowest yields were observed at the same treatment time but with a 25 kV applied voltage in LHA6 (25.39 ± 0.53 mg/g_d.m._) and LHN6 (14.27 ± 0.53 mg/g_d.m._) samples. The maximum difference between the sample groups was observed at 25 kV and 3 min, where the protein yield was 1.78× higher in the LHA6 sample, compared to the LHN6. In general, for all LHA and LHN samples, the protein yield was 7.30 – 17.85 times lower compared to the control sample extracted by the phenolic extraction method (254.67 ± 8.85 mg/g_d.m._). Moreover, the results of US extractions were considerably higher in comparison to the results of HVED extraction. In particular, the highest difference between the two extraction techniques was observed between the LUDI6 and LHN6 samples, where a 6.78× higher protein yield was recorded in the LUDI6 sample.

Through statistical data processing, it was observed that in both HVED groups of samples, neither the input variable (voltage and treatment time) nor their mutual interaction had a statistically significant impact on the protein yield, *p >* 0.05 (Table 4). However, by optimizing the HVED extraction parameters for both gases, a voltage of 20 kV and a treatment time of 3 min proved to be optimal regarding protein yield.

**Table 4:** Statistical significance for protein yield. MANOVA statistically processes the variability of each input parameter, their mutual interactions, and quadratic interactions of treatment time on the output values of LHA and LHN samples.

Sample group		Main effects		Interactions	
		A: Voltage	B: Treatment time	AB	BB
*p*	LHA	0.92	0.77	0.36	0.92
	LHN	0.33	0.54	0.21	0.81

Where A determines Voltage and B stands for Treatment time. The p-values > 0.05, indicating that they are not significantly different from zero at the 95.0% confidence level.

### 3.2 RuBisCO determination

#### 3.2.1 RuBisCO determination after US extraction

**Fig. 3:**
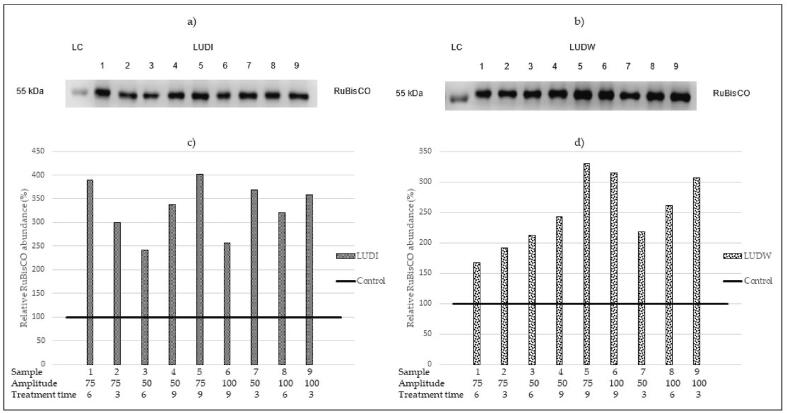
Immunoblotting analysis of RuBisCO after US extractions in a) LUDI and b) LUDW samples. Figures c) and d) represent the relative abundance of LUDI samples (c), and LUDW samples (d) in comparison to the control sample. The applied amplitude (%) and treatment time (min) are listed below each sample.

RuBisCO protein was detected by immunoblotting in all sugar beet leaf extracts obtained by US extraction (Fig. 3). In general, a higher abundance of RuBisCO enzyme was observed in US-treated samples, compared to the control sample. Specifically, 2.41 – 4.01× higher abundance of RuBisCO enzyme was recorded in LUDI samples, and 1.67 – 3.30× in LUDW samples. Among the US extraction method, the lowest relative abundance of RuBisCO was recorded in LUDI3 and LUDW1 samples, whereas the highest abundance was observed in LUDI5 and LUDW5 samples with 75% applied amplitude and 9 min treatment time. Furthermore, at 75% amplitude, the relative abundance of the RuBisCO enzyme increased with longer treatment time in LUDI samples. At 50% and 100% amplitude, no increasing trend was recorded. In contrast, at 100% amplitude, the abundance of the target enzyme decreased with a longer treatment time. Onwards, at a treatment time of 6 min, with higher amplitude, the increase in the abundance of RuBisCO enzyme in the LUDW samples was observed. Immunoblotting results were used for screening for the samples with the highest abundance of the target enzyme, which were quantified using liquid chromatography coupled with the mass spectrometry (LC-MS) method, and their concentration amounted to 224.84 ± 0.45 µg/mL for LUDI5, and 202.96 ± 2.90 µg/mL for the LUDW5 sample. Although no major difference in concentrations was observed among US-treated samples, the difference between US and HVED-treated samples was not negligible (2.76 – 19.00× higher in US-treated samples).

#### 3.2.2 RuBisCO determination after HVED extraction

**Fig. 4:**
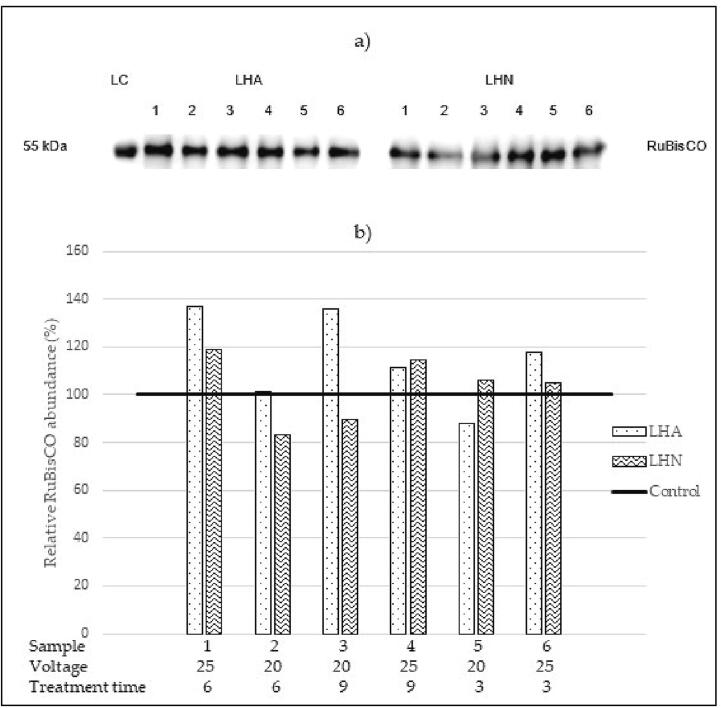
a) immunoblotting analysis of rubisco after hved extractions. figure b) represents the relative abundance of LHA and LHN samples in comparison to the control sample. The applied voltages (kv) and treatment time (min) are listed below each sample.

Relative protein abundances for HVED-treated samples are shown in Fig. 4. In comparison to the control sample, the HVED-treated samples mostly showed a higher abundance of RuBisCO. The exceptions were samples LHA5, LHN2, and LHN3, where protein abundance was lower than in the control sample by 10.42 – 16.77%. Looking at each sample group separately, LHA1 and LHN1 samples showed the highest protein abundance within their respective groups. However, the amount of RuBisCO in the LHA1 sample was higher than in the LHN1 sample. Given that the applied voltage (25 kV) and the treatment time (6 min) were the same, the difference was most likely due to the different impacts of the applied gas (argon or nitrogen). Furthermore, at the same voltages and treatment times, higher levels of RuBisCO protein were recorded in most samples treated with argon (66.67%) compared to those treated with nitrogen. Within each group of samples, the samples with the highest RuBisCO's abundance were quantified by the LC-MS method. The concentration of RuBisCO in the LHA1 sample (81.32 ± 0.73 µg/mL) was 6.87× higher compared to the LHN1 sample (11.83 ± 0.02 µg/mL).

### 3.3 pH

#### 3.3.1 pH after US extraction

The results of pH values for ultrasonically treated samples were previously published in Dukić et al. [19]. Results did not differ significantly between individual groups and ranged from 6.98 ± 0.19 to 7.12 ± 0.19 for LUDI and from 6.97 ± 0.19 to 7.12 ± 0.08 for LUDW samples.

#### 3.3.2 pH after HVED extraction

**Fig. 5:**
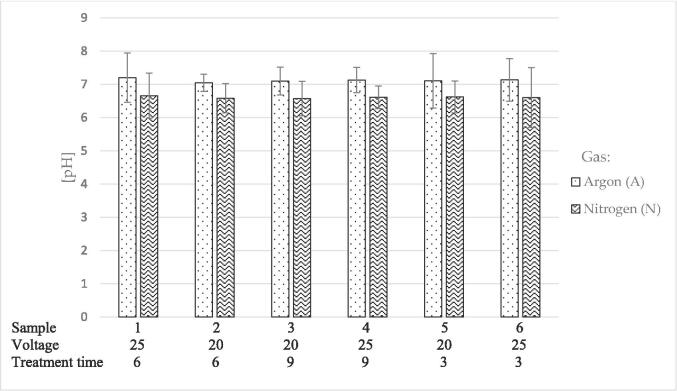
pH values for hved-treated LHA and LHN samples. The applied voltages (kv) and treatment time (min) are listed below each sample. Results were presented as mean value of replicates ± standard deviation.

Given that the solubility of proteins largely depends on the pH range, the pH values after HVED treatments were measured. The obtained values are shown in Fig. 5. In relation to the LHA samples (7.047 ± 0.26 – 7.202 ± 0.74), lower pH values were recorded in LHN samples (6.571 ± 0.52 – 6.658 ± 0.68).

## 4 Discussion

The use of non-thermal extraction techniques and the selection of green solvents contribute to the cost reduction, and promotes a more environmentally acceptable approach compared to conventional extraction methods [64], [65], [66]. Therefore, the valorization of food waste using novel techniques and green solvents is on the rise. In this paper, the impact of various US and HVED extraction parameters on the amount of total soluble proteins, particularly RuBisCO, extracted from sugar beet leaves was investigated. As expected, the protein yield of the control sample exceeded the yields of the US (2.63 – 9.83×) and HVED-treated samples (7.30 – 17.85×). Phenol extraction (control sample) is the gold standard chemical method for protein extraction due to its capacity of obtaining high-purity proteins. By preventing proteolytic activity during extraction, minimal protein degradation was ensured [59]. Furthermore, as a result of the dissociation effect, molecular interactions between proteins and some other molecules such as phenolic compounds, nucleic acids, etc., were reduced [67]. All of the above corroborates the larger amounts of proteins observed in the control sample in comparison to the US and HVED-treated samples. However, considering the rather long extraction time (> 24 h) and the use of toxic chemicals (phenol, methanol, β-mercaptoethanol, PMSF) [59], there is no question that this extraction method is economically and environmentally less acceptable in comparison to US and HVED extraction in terms of scaling up the extraction process to an industrial scale. When comparing these two alternative methods, the US method proved to be by far the more efficient method for protein extraction. Higher protein yield in US samples can be attributed to the high shear and mass transfer effects caused by acoustic cavitation [68]. This is consistent with previous studies with the Lowry method for protein quantification. In particular, for the same plant material and in comparison, with the results of US extraction [19], HVED treatment proved to be a less efficient method for protein extraction. Specifically, in dry US-treated samples obtained protein yields were 1.91 – 7.51 folds higher (66.72 ± 4.56 – 107.20 ± 9.23 mg/g_d.m._), than in LHA and LHN samples (14.27 ± 0.53 – 34.89 ± 1.59 mg/g_d.m._). Regardless of the different quantification methods, the highest protein yields were recorded under the same US extraction parameters. However, lower protein yields were reported in US samples determined by the Bradford method. Protein quantification methods are partially dependent on amino acid composition [69] and significant differences in concentration were reported using identical samples [70]. But, for plant protein determination, the Bradford assay is more suitable [59]. Contrary to the conducted research and compared to applied pre-treatments such as the US and pulsed electric field (PEF), the application of HVED showed the highest protein yield in wine shoot samples [71]. Similar results were also recorded in olive kernel samples, where at the same input of energy of 18 kJ/kg, the highest protein yield was observed with HVED as a pre-treatment [72]. In addition to the above, sesame cake samples pre-treated with HVED and PEF showed a higher protein yield compared to the control sample (without pre-treatment) [73]. These higher protein yields were attributed to the combined effect of the mechanical and hydrodynamic effects of HVED treatment [71].

Although a change in total protein amount was observed when solvents were used at different temperatures under the same US extraction conditions (Fig. 1), no significant differences in RuBisCO yield were observed between LUDI5 and LUDW5 samples. On the other hand, significant differences were observed between US and HVED-treated samples. Higher yields of proteins can be attributed to cavitation, mechanical, and thermal effects of US which consequently affect the expression level of genes related to metabolic synthesis pathways. Increased expression mainly occurs as a defensive response to stressful environmental conditions [74]. Furthermore, the formation of free radicals such as **·**OH radicals, **·**O radicals, and hydrogen peroxide (H_2_O_2_), slightly affects the change in pH value and consequently the yields of different enzymes in the samples. Namely, at a frequency of 20 kHz, the formation of OH radicals in the aqueous medium as a result of the dissociation of water vapor and oxygen inside the cavitation bubble [75], [76], [77], leads to increased pH values in the samples [78]. In general, the pH values of the samples are extremely important for the solubility of RuBisCO, which consequently affects its yield. In general, the lowest solubility of a protein is in the region of its isoelectric point (pI). In this area, due to the zero net charge, protein–protein interactions increase, and protein-water interactions decrease. As a result, the protein molecules are close enough to each other and they aggregate. By moving away from the pI region, the net charge of the protein changes and a greater number of molecules interact with water, which increases the solubility of the protein [79]. In US-treated samples, the use of solvents at different temperatures did not significantly affect the change in pH, and consequently, the solubility of the target enzyme. On the other hand, differences in the abundance of RuBisCO were observed due to the use of different gases during HVED extraction. Specifically, a lower abundance was observed in samples treated with nitrogen, compared to those treated with argon (Fig. 4). Nitrogen is a chemically inert gas, and as such could be a preferable choice for the extraction of bioactive compounds [80]. On the other hand, as a result of the electric discharge, nitrogen molecules dissociate into nitrogen radicals, which, in the presence of oxygen and oxygen radicals, recombine to form nitrate and nitrite compounds [81], the presence of which consequently leads to a drop in pH during HVED extraction. Considering the pI of RuBisCO, which ranges from 6.10 to 6.73 [33], [82], the pH of LHN samples got into the specified pI range. The lack of electrostatic repulsion in the pI region leads to protein aggregation resulting in lower protein solubility [83]. As a consequence of lower solubility due to a decrease in pH (Fig. 5), the abundance of RuBisCO in samples treated with nitrogen was lower than with argon as mentioned before (Fig. 4). The formation of OH^–^ ions in water following an electric discharge with applied argon, leads to an increase in pH [84]. By increasing the pH and moving away from the pI point, the solubility of RuBisCO increased. Because ultrasound has rarely been used for enzyme extraction and HVED is a relatively new extraction technique, there is a lack of research and results related to the extraction of RuBisCO from sugar beet leaves or other plant sources. Compared to some other methods of extraction, such as extraction with biocompatible ionic liquids (IL), i.e., IL derived from choline and glycine-betaine analogues, US and HVED extractions gave lower yields of RuBisCO [85]. Specifically, by extracting RuBisCO from spinach using choline acetate ([Ch][Ac]) and choline chloride ([Ch]Cl), a yield of 10.92 – 10.57 mg/g biomass was achieved. The stated results are multiple times higher than the results from our study, obtained by US and HVED extraction of sugar beet leaves. Higher RuBisCO yield (27%) was also observed in duckweed samples, where mechanical extraction and isolation by precipitation and coagulation was conducted [86]. Lower yields can be attributed to the presence of phenolic compounds, chlorophyll, and some other compounds, which make the extraction of RuBisCO challenging [36]. In general, currently available extraction methods are not sufficiently efficient and selective, resulting in lower amounts of RuBisCO [85]. Furthermore, apart from the extraction conditions, the RuBisCO yield also depends on the sample purification methods [87]. Specifically, at the industrial level, after thermal precipitation (50 °C, 30 min) and solubilization with surfactants, a 0.67% yield of RuBisCO from sugar beet leaves was recorded [88]. In laboratory conditions, after enzymatic extraction and precipitation, a 0.3% yield of RuBisCO was observed in Arabidopsis florets leaf samples [89].

In future research, the yield of RuBisCO could potentially be increased by the combined application of US or HVED and natural deep eutectic solvents (NADES). The use of such solvents should increase the solubility, stability, and biological activity of enzymes [90]. Furthermore, the application of other non-thermal techniques such as enzyme-assisted extraction would increase the possibilities for extraction of RuBisCO and other enzymes.

## 5 Conclusions

Sugar beet leaves, which are waste from the sugar industry, are a good and sustainable source of high-value compounds such as proteins. In this paper, it was demonstrated that proteins can be extracted in a very efficient and economical way, by using a green solvent and non-thermal extraction techniques. In general, US extraction was found to be more efficient than HVED extraction for total protein extraction and for obtaining the highest RuBisCO yields. Although different parameters used for the US extractions exhibited similar results, the highest yield of RuBisCO was observed in the sample with room-temperature water as the extraction solvent. Considering the nutritional value of RuBisCO and of proteins in general, it is more and more certain that they will find its application in the manufacture of functional products such as desserts, spreads, beverages, etc. The development of new products from waste would not only reduce the problem of food shortage in the world, but also reduce the harmful impact on the environment. Due to all the above, the application of purified RuBisCO extract in the future of food production is promising.

## Funding

This research was funded by the Partnership for Research and Innovation in the Mediterranean Area PRIMA H2020 GA2032: “FunTomP—Functionalized Tomato Products”. The work of doctoral student Josipa Dukić has been fully supported by the “Young researchers’ career development project—training of doctoral students” of the Croatian Science Foundation (DOK-2021-02).

## CRediT authorship contribution statement

**Josipa Dukić:** Conceptualization, Methodology, Software, Formal analysis, Investigation, Data curation, Writing – original draft, Writing – review & editing, Visualization, Funding acquisition. **Karla Košpić:** Methodology, Software, Formal analysis, Investigation, Writing – original draft, Writing – review & editing. **Vanja Kelava:** Formal analysis, Investigation, Writing – original draft, Writing – review & editing. **Renata Mavrić:** Investigation. **Marinela Nutrizio:** Writing – review & editing, Visualization. **Biljana Balen:** Validation, Supervision. **Ana Butorac:** Methodology, Validation. **Mecit Halil Oztop:** Resources, Supervision, Project administration. **Anet Režek Jambrak:** Conceptualization, Methodology, Validation, Resources, Supervision, Project administration, Funding acquisition.

## Declaration of Competing Interest

The authors declare that they have no known competing financial interests or personal relationships that could have appeared to influence the work reported in this paper.
