# Supplementary material for: Alternative methods for RuBisCO extraction from sugar beet waste: A comparative approach of ultrasound and high voltage electrical discharge
Source: Ultrason Sonochem. 2023 Jul 27;99:106535. doi: 10.1016/j.ultsonch.2023.106535 (PMC10410599; doi:10.1016/j.ultsonch.2023.106535)
Supplement: Supplementary data 1 [file mmc1.docx]

**Figure S1.** Calibration curve for protein quantification.

**Figure S2.** Calibration curve for TFQGPPHGIQVER peptide quantification. Relative concentrations are expressed in ng/mL.

**Figure S3.** Calibration curve for AQAETGEIK peptide quantification. Relative concentrations are expressed in ng/mL.
